# Knowledge, attitudes, and proactive practices regarding PICC in gastrointestinal cancer: a mediation analysis of a patient empowerment attempt

**DOI:** 10.3389/fmed.2025.1675258

**Published:** 2026-01-05

**Authors:** Min Wei, Cailian Liu, Shuyuan Zhuang

**Affiliations:** Department of Oncology, Peking University Cancer Hospital, Inner Mongolia Hospital, Hohhot, China

**Keywords:** gastrointestinal cancer, PICC, knowledge, attitudes, and practices, self-care ability, mediation analysis, proactive practices, patient empowerment

## Abstract

**Objective:**

To examine the knowledge, attitudes, and practices (KAP) related to the use and self-care of peripherally inserted central catheters (PICC) among gastrointestinal cancer patients.

**Methods:**

This cross-sectional study was conducted between January and July 2024 at Inner Mongolia Hospital and Peking University Cancer Hospital. A questionnaire was used to collect self-reported KAP data. Achieving scores above 70% of the maximum in each section indicated adequate knowledge, positive attitudes, and proactive practices. A mediation analysis was performed to examine the relationships among KAP dimensions.

**Results:**

A total of 444 valid cases were included. Of these, 305 (68.69%) were male, and 338 (76.13%) had undergone a single PICC placement. The knowledge, attitude, and practice scores were 11.27 ± 3.69 (possible range: 0–14; 80.50%), 30.48 ± 3.22 (possible range: 8–40; 76.20%), and 31.32 ± 5.38 (possible range: 8–40; 78.30%), respectively, indicating good KAP. Path analysis revealed that duration of illness (β = -1.04, *P* = 0.001) and self-care ability (β = -1.08, *P* = 0.01) had a direct negative influence on knowledge. Knowledge (β = 0.27, *P* < 0.001) and higher education (β = 0.36, *P* = 0.021) had a positive influence on attitude, while type of tumor (β = -0.31, *P* = 0.041) had a direct negative influence.

**Conclusions:**

Patients demonstrated sufficient knowledge, generally positive attitudes, and proactive practices regarding PICC. Based on the KAP theoretical framework, targeted educational interventions to improve patients’ knowledge and self-care abilities and positively shape their attitudes may further enhance PICC management and optimize patient outcomes by promoting patient empowerment. These findings highlight the importance of integrating self-care training into patient discharge protocols.

## 1 Introduction

Gastrointestinal cancers comprise a diverse group of malignancies affecting organs such as the esophagus, stomach, liver, pancreas, colon, and rectum, as well as less common sites like the gallbladder, small intestine, and anus (1). These cancers account for 26% of global cancer cases and are responsible for 35% of all cancer-related deaths, with an estimated 4.8 million new cases and 3.4 million related deaths worldwide (2). Current treatment strategies for gastrointestinal cancers primarily involve surgery, endoscopy, radiotherapy, chemotherapy, targeted therapy, and immunotherapy (3, 4). These treatments often necessitate long-term venous access for administering chemotherapy, nutritional support, and blood transfusions. Effectively managing the care requirements of patients undergoing these treatments is critical for enhancing their quality of life and achieving optimal treatment outcomes.

Peripherally inserted central catheters (PICCs) have become indispensable in the care of gastrointestinal cancer patients. They offer reliable, long-term venous access with a lower complication risk compared to conventional venous access methods (5). PICCs are particularly beneficial for delivering chemotherapy and providing parenteral nutrition, especially for patients who may struggle with enteral nutrition due to the impact of cancer on their digestive system, especially in advanced stages of the disease (6, 7). In addition, tumor-related issues such as appetite loss, malabsorption, and gastrointestinal obstructions can hinder adequate nutrient intake, while treatments like surgery, radiotherapy, and chemotherapy further compromise the body’s ability to absorb nutrients (8).

Proper planning and management of long-term venous access, especially for devices such as PICCs, are essential for minimizing complications, improving treatment outcomes, and maintaining patient safety. PICCs are commonly utilized when intravenous therapy is required for more than 6 days or when peripheral access is difficult, and their success depends heavily on diligent maintenance and daily assessment (9, 10). Well-structured planning ensures the selection of the most suitable venous access device for each patient, accounting for their clinical needs, expected treatment duration, and lifestyle factors. Collaboration between healthcare teams and patients is vital to choosing the right device and implementing evidence-based maintenance protocols, which reduces the risk of infection, device malfunction, and venous depletion (9, 11). PICC dressing changes should occur every 7–10 days or sooner if the dressing becomes loose, wet, or soiled, aligning with safety guidelines. Daily assessment of the dressing and exit site is critical, including checking for signs of infection (redness, swelling, discharge) or dressing compromise. While clinical staff are responsible for insertion and major interventions, only the patient or their caregiver can reliably perform daily checks outside the healthcare setting, making patient education and engagement indispensable. This enhances early detection of complications and ensures timely intervention, greatly reducing risks associated with long-term venous access (10).

A knowledge, attitudes, and practices (KAP) survey serves as a valuable tool in health research, helping to assess a population’s understanding, beliefs, and behaviors related to a specific health issue. The KAP model is based on the premise that knowledge influences attitudes, which subsequently affect behaviors (12, 13). In the context of PICC use, the KAP framework can highlight gaps in patients’ understanding and behaviors that may lead to complications. Although previous studies have explored the use of PICC in cancer patients, they have not comprehensively addressed KAP specifically related to PICC in gastrointestinal cancers (14). Moreover, most existing studies focus on healthcare providers rather than patients, leaving a gap in understanding how patient-level knowledge and behaviors impact PICC outcomes (7, 15, 16).

Despite the increasing use of PICCs in cancer care, limited research has examined how patients’ knowledge and attitudes influence their PICC-related practices, particularly in the gastrointestinal cancer population. Therefore, this study aimed to assess the KAP of gastrointestinal cancer patients concerning PICC management.

## 2 Material and methods

### 2.1 Study design and patients

This cross-sectional study enrolled gastrointestinal cancer patients between January and July 2024 in the Gastrointestinal Oncology Department and the outpatient clinic of Inner Mongolia Hospital, Peking University Cancer Hospital. The study was approved by the Ethics Review Committee of Inner Mongolia Hospital, Peking University Cancer Hospital (Approval No.: WZ202405), and informed consent was obtained from all participants.

Inclusion criteria: (1) Patients with gastrointestinal cancer who had undergone PICC line placement; (2) Age between 18 and 80 years. Exclusion criteria: (1) Patients with cognitive or psychiatric disorders that impaired their ability to participate; and (2) Patients who declined to participate.

### 2.2 Questionnaire

The questionnaire was developed by the investigators based on the “*Expert Consensus on Venous Catheter Maintenance*” (17). The items were developed based on the expert consensus to cover the basic knowledge on PICC, the proper attitudes to maintain when having a PICC, and the optimal practice in maintaining a PICC. Content was validated by experts in infusion and PICC use. A pilot survey involving 27 participants yielded a reliability coefficient of 0.7415 (0.6800 for knowledge, 0.6628 for attitudes, and 0.7274 for practice), indicating acceptable internal consistency.

The final questionnaire (Supplementary material), written in Chinese, consists of four sections: demographic information, knowledge dimension, attitude dimension, and practice dimension. The knowledge dimension comprises 14 items, with correct answers receiving 1 point and incorrect or unclear answers receiving 0 points, resulting in a total score range of 0–14. The attitude dimension contains 8 items measured on a five-point Likert scale, with a total score range of 8–40. The practice dimension consists of 8 questions, scored from 1 (never) to 5 (always), with a total score range of 8–40. Self-care was evaluated using the question “What is your level of self-care ability?”, with three possible choices (fully able, partially able, and unable). Achieving scores above 70% of the maximum in each section indicated adequate knowledge, positive attitudes, and proactive practices (18).

### 2.3 Questionnaire distribution and quality control

The questionnaire was distributed through convenience sampling to patients via QR code scanning on their mobile phones. After completing the survey, two research assistants, who had completed specialized PICC training certified by the Autonomous Region Nursing Association and were familiar with the questionnaire content, provided face-to-face clarification for any patient concerns.

To ensure data integrity, questionnaires were deemed invalid if they met any of the following criteria: (1) completion time of less than 90 s; (2) inaccurately provided basic information; and (3) incorrect answers to trap questions designed to ensure respondent attentiveness. Specifically, the trap question was: “If the catheter is found to be dislodged, it can be adjusted by oneself.” It was presented in two versions with reversed option orders: one as “(a) correct (b) incorrect (c) unclear,” and the other as “(a) incorrect (b) correct (c) unclear.” Respondents who gave contradictory answers to the two versions were considered to have failed the attention check.

### 2.4 Sample size calculation

The sample size calculation was performed based on a desired confidence level of 95% and a margin of error of 5%. Using the formula for sample size calculation for proportions (19):

*N* = (Z^∧^2 * *p* * (1–*p*))/e^∧^2

where:

N is the required sample size,

Z is the Z-score corresponding to a 95% confidence level (1.96),

p is the estimated proportion of the population (assumed to be 0.5 for maximum variability),

e is the margin of error (0.05).

Substituting these values into the formula yields a required sample size of 384 participants. Considering 20% of excluded questionnaires, a total of 480 questionnaires had to be collected.

### 2.5 Statistical analysis

Data analysis was conducted using STATA 17.0 (Stata Corporation, College Station, TX, United States). Quantitative data were presented as mean and standard deviation ± standard deviation and were tested for normal distribution using the Kolmogorov-Smirnov test and for homogeneity of variance using Levene’s test. For group comparisons, Student’s *t*-test (comparison of two groups/levels) or ANOVA (comparison of more than two groups/levels) were used for normally distributed data, while the Mann-Whitney U-test (comparison of two groups/levels) or the Kruskal-Wallis H-test (comparison of more than two groups/levels) were applied for non-normally distributed data. Categorical data were expressed as frequency (percentage). Spearman correlation analysis was used to evaluate the relationships between knowledge, attitude, and practice scores. Path analysis was performed to test the following hypotheses: (1) specific factors (education, duration of illness, self-care ability, etc.) would influence knowledge, attitudes, and practices (12, 20). (2) knowledge would influence attitudes; (3) knowledge would influence practices; (4) attitudes would influence practices. Model fit was assessed using Root Mean Square Error of Approximation (RMSEA) (< 0.08 is good), Standardized Root Mean Square Residual (SRMR) (< 0.08 is good), Tucker-Lewis Index (TLI) (> 0.8 is good), and Comparative Fit Index (CFI) (> 0.8 is good). A two-sided *p* <0.05 was considered statistically significant.

## 3 Results

### 3.1 Demographic information

Table 1 presents the characteristics of the participants. This study collected 635 questionnaires. After excluding five questionnaires with contradictory responses, 29 with a completion time of < 60 s, four because reported age was < 18 or impossible, and 161 due to failure to answer the trap questions correctly. Therefore, 444 valid questionnaires were included for analysis. Among these, 305 (68.69%) were male, with a mean age of 62.22 ± 10.60 years (range: 21–87). The knowledge, attitude, and practice scores were 11.27 ± 3.69 (possible range: 0–14; 80.50%), 30.48 ± 3.22 (possible range: 8–40; 76.20%), and 31.32 ± 5.38 (possible range: 8–40; 78.30%), respectively, indicating good KAP (Table 1).

**TABLE 1:** Basic characteristics.

*N* = 444	*N* (%)	Knowledge score		Attitude score		Practice score	
		Mean ± SD	*P*	Mean ± SD	*P*	Mean ± SD	*P*
Total score		11.27 ± 3.69		30.48 ± 3.22		31.32 ± 5.38	
Age (years)			0.243		0.156		0.236
< 65	219 (49.32)	11.52 ± 3.54		30.74 ± 3.59		31.63 ± 5.56	
≥ 65	225 (50.68)	11.02 ± 3.82		30.22 ± 2.79		31.01 ± 5.18	
Gender			0.731		0.696		0.410
Male	305 (68.69)	11.26 ± 3.70		30.58 ± 3.33		31.47 ± 5.33	
Female	139 (31.31)	11.29 ± 3.68		30.26 ± 2.95		30.97 ± 5.47	
Residence			0.825		0.324		0.171
Urban	275 (61.94)	11.27 ± 3.73		30.62 ± 3.14		31.52 ± 5.31	
Rural	129 (29.05)	11.27 ± 3.73		30.35 ± 3.48		31.32 ± 5.67	
Suburban	40 (9.01)	11.22 ± 3.33		29.95 ± 2.83		29.87 ± 4.70	
Education			0.628		**0.001**		**0.006**
Primary school or below	70 (15.77)	11.32 ± 3.16		30.82 ± 3.35 ^ac^		31.45 ± 5.59 ^ab^	
Middle school	197 (44.37)	11.13 ± 3.99		29.96 ± 3.07 ^a^		30.54 ± 5.50 ^a^	
High school/technical school	113 (25.45)	11.46 ± 3.56		30.39 ± 2.77 ^a^		31.43 ± 5.00 ^ab^	
Associated degree/bachelor’s degree or above	64 (14.4)	11.31 ± 3.54		31.84 ± 3.83 ^bc^		33.34 ± 4.92 ^b^	
Monthly household income (RMB)			**0.008**		0.873		0.202
< 2,000	102 (22.97)	11.22 ± 3.31 ^a^		30.54 ± 3.60		32.16 ± 5.72	
2,000–5,000	199 (44.82)	10.78 ± 4.16 ^a^		30.41 ± 2.93		30.92 ± 5.49	
> 5,000–10,000	144 (32.43)	11.97 ± 3.11 ^b^		30.54 ± 3.34		31.26 ± 4.90	
Type of tumor			0.649		**0.015**		0.294
Esophageal cancer	53 (11.94)	11.52 ± 3.46		30.52 ± 3.60 ^ab^		30.69 ± 6.33	
Gastric cancer	128 (28.83)	11.46 ± 3.22		31.18 ± 3.29 ^a^		31.96 ± 5.33	
Colorectal cancer	176 (39.64)	11.42 ± 3.52		30.27 ± 3.02 ^ab^		30.90 ± 4.89	
Other tumor	87 (19.59)	10.52 ± 4.65		29.85 ± 3.12 ^b^		31.58 ± 5.69	
Duration of illness (years)			**0.001**		**0.048**		0.295
< 1	283 (63.74)	11.61 ± 3.17 ^a^		30.55 ± 3.13 ^ab^		31.34 ± 5.36	
1–3	128 (28.83)	11.58 ± 3.36 ^a^		30.65 ± 3.39 ^a^		31.57 ± 5.51	
> 3	33 (7.43)	7.151 ± 5.99 ^b^		29.24 ± 3.10 ^b^		30.15 ± 4.89	
Level of self-care ability			**0.017**		0.160		0.285
Fully self-care	348 (78.38)	11.58 ± 3.26		30.60 ± 3.21		31.44 ± 5.35	
Partial self-care	96 (21.62)	10.14 ± 4.80		30.04 ± 3.22		30.85 ± 5.47	
Number of PICC insertions you underwent			**0.004**		0.514		0.600
1	338 (76.13)	11.71 ± 3.13 ^a^		30.53 ± 3.18		31.19 ± 5.36	
2	69 (15.54)	9.927 ± 4.87 ^ab^		30.02 ± 3.51		31.50 ± 5.58	
≥ 3	37 (8.33)	9.783 ± 4.85 ^b^		30.86 ± 2.95		32.08 ± 5.17	
Duration of your most recent PICC placement (month)			0.620		0.761		**0.007**
< 1	106 (23.87)	11.25 ± 3.48		30.43 ± 3.07		32.72 ± 5.25 ^a^	
1–3	152 (34.23)	11.38 ± 3.30		30.68 ± 3.50		31.25 ± 5.41 ^ab^	
3–6	112 (25.23)	11.09 ± 4.08		30.5 ± 3.43		30.90 ± 5.17 ^b^	
>6	74 (16.67)	11.32 ± 4.15		30.12 ± 2.43		30.08 ± 5.44 ^b^	

*^abc^*Values with different superscript letters are significantly (*P* < 0.05) different according to the Bonferroni Method. Bold values represent statistically significant results (*P* < 0.05).

### 3.2 Distribution of responses to knowledge, attitudes, and practices

Table 2 presents the distribution of the responses to the knowledge items. In the knowledge domain, the three items most frequently marked as “unsure” were: “PICC patients with hyperlipidemia are at risk of developing thrombosis” (K11) at 20.72%, “Irritant medications may be one of the causes of phlebitis in PICC patients” (K5) at 18.92%, and “Thrombosis can cause pain, swelling of the affected limb, and difficulty in movement” (K13) at 17.79% (Table 2).

**TABLE 2:** Knowledge distribution.

Knowledge, N (%)	True	False	Unsure
PICC, also known as a Peripherally Inserted Central Catheter, is inserted through a peripheral vein into the central venous system.	276 (62.16)	57 (12.84)	111 (25)
PICC can be left in the blood vessel for a long time, facilitating continuous infusion and protecting the veins.	351 (79.05)	34 (7.66)	59 (13.29)
During bathing, protective film should be used to cover the PICC and it should be replaced promptly.	400 (90.09)	4 (0.9)	40 (9.01)
If redness, swelling, pain, and purulence occur at the puncture site after PICC placement, medical attention should be sought promptly.	409 (92.12)	2 (0.45)	33 (7.43)
Irritant medications may be one of the causes of phlebitis in PICC patients.	353 (79.5)	7 (1.58)	84 (18.92)
To prevent occlusion, PICC needs to be flushed with heparin weekly at the hospital.	352 (79.28)	22 (4.95)	70 (15.77)
If the catheter is obstructed, it should be removed directly.	59 (13.29)	294 (66.22)	91 (20.5)
To prevent catheter displacement, the arm with the PICC should avoid intense activities and external impact.	389 (87.61)	10 (2.25)	45 (10.14)
If the catheter becomes displaced, it can be adjusted by oneself.	4 (0.9)	389 (87.61)	51 (11.49)
When there is seepage or bleeding from the PICC site, no action is required.	52 (11.71)	321 (72.3)	71 (15.99)
PICC patients with hyperlipidemia are at risk of developing thrombosis.	337 (75.9)	15 (3.38)	92 (20.72)
To prevent thrombosis, gentle exercises such as fist clenching and stretching can be done with the arm that has the PICC.	381 (85.81)	5 (1.13)	58 (13.06)
Thrombosis can cause pain, swelling of the affected limb, and difficulty in movement.	361 (81.31)	4 (0.9)	79 (17.79)
The timing of PICC removal is determined by the doctor and should not be done by oneself.	392 (88.29)	4 (0.9)	48 (10.81)

Table 3 presents the distribution of the responses to the attitude items. For attitudes, 38.51% expressed concern about being unable to properly care for the PICC (A6), 31.76% agreed that PICC insertion affected their daily life (A5), and 12.61% lacked confidence in identifying PICC-related complications in a timely manner (A7) (Table 3).

**TABLE 3:** Attitude distribution.

Attitudes, N (%)	Strongly agree	Agree	Neutral	Disagree	Strongly disagree
I believe that PICC can alleviate the pain of frequent injections associated with ordinary infusion.	179 (40.32)	214 (48.2)	41 (9.23)	6 (1.35)	4 (0.9)
I believe that regular maintenance of the PICC is very important.	216 (48.65)	204 (45.95)	19 (4.28)	3 (0.68)	2 (0.45)
I believe that maintaining personal hygiene is very important for PICC maintenance.	219 (49.32)	202 (45.5)	20 (4.5)	1 (0.23)	2 (0.45)
I believe that avoiding intense activities helps prevent PICC displacement and displacement.	219 (49.32)	203 (45.72)	14 (3.15)	5 (1.13)	3 (0.68)
I believe that PICC placement has affected my normal life.	82 (18.47)	141 (31.76)	111 (25)	92 (20.72)	18 (4.05)
I am concerned that I might not be able to care for the PICC properly.	94 (21.17)	171 (38.51)	112 (25.23)	54 (12.16)	13 (2.93)
I am confident in my ability to recognize PICC-related complications in a timely manner.	106 (23.87)	155 (34.91)	115 (25.9)	56 (12.61)	12 (2.7)
I believe that the hospital should organize educational programs about PICC.	199 (44.82)	210 (47.3)	28 (6.31)	2 (0.45)	5 (1.13)

Table 4 presents the distribution of the responses to the practice items. In terms of practices, 10.14% never avoided bathing or swimming after receiving a PICC (P1) and never took the initiative to learn about PICC catheterization (P8). Additionally, 16.22% rarely checked for blood return in the catheter (P7), and 12.84% rarely inspected the PICC for damage (P6) (Table 4).

**TABLE 4:** Practice distribution.

Practices, N (%)	Always	Often	Sometimes	Rarely	Never
I avoid taking baths or swimming while the PICC is in place.	163 (36.71)	181 (40.77)	27 (6.08)	28 (6.31)	45 (10.14)
I regularly visit the hospital for PICC maintenance.	184 (41.44)	183 (41.22)	60 (13.51)	13 (2.93)	4 (0.9)
I promptly replace the protective film if I notice it is curled, peeling, or loose.	178 (40.09)	175 (39.41)	72 (16.22)	14 (3.15)	5 (1.13)
I avoid lifting heavy objects with the arm that has the PICC.	194 (43.69)	177 (39.86)	43 (9.68)	10 (2.25)	20 (4.5)
I avoid compressing the catheter.	188 (42.34)	182 (40.99)	51 (11.49)	10 (2.25)	13 (2.93)
I check the PICC daily for any damage.	143 (32.21)	146 (32.88)	82 (18.47)	57 (12.84)	16 (3.6)
I check daily for blood return in the catheter.	129 (29.05)	134 (30.18)	91 (20.5)	72 (16.22)	18 (4.05)
I actively learn about PICC catheterization knowledge.	109 (24.55)	102 (22.97)	118 (26.58)	70 (15.77)	45 (10.14)

### 3.3 Correlation analysis

Supplementary Table 1 presents the correlation analyses. Correlation analysis revealed significant positive relationships between knowledge and attitudes (*r* = 0.2468, *P* < 0.001), knowledge and practices (*r* = 0.2432, *P* < 0.001), and attitudes and practices (*r* = 0.4132, *P* < 0.001) (Supplementary Table 1).

### 3.4 Path analysis

**FIGURE 1:**
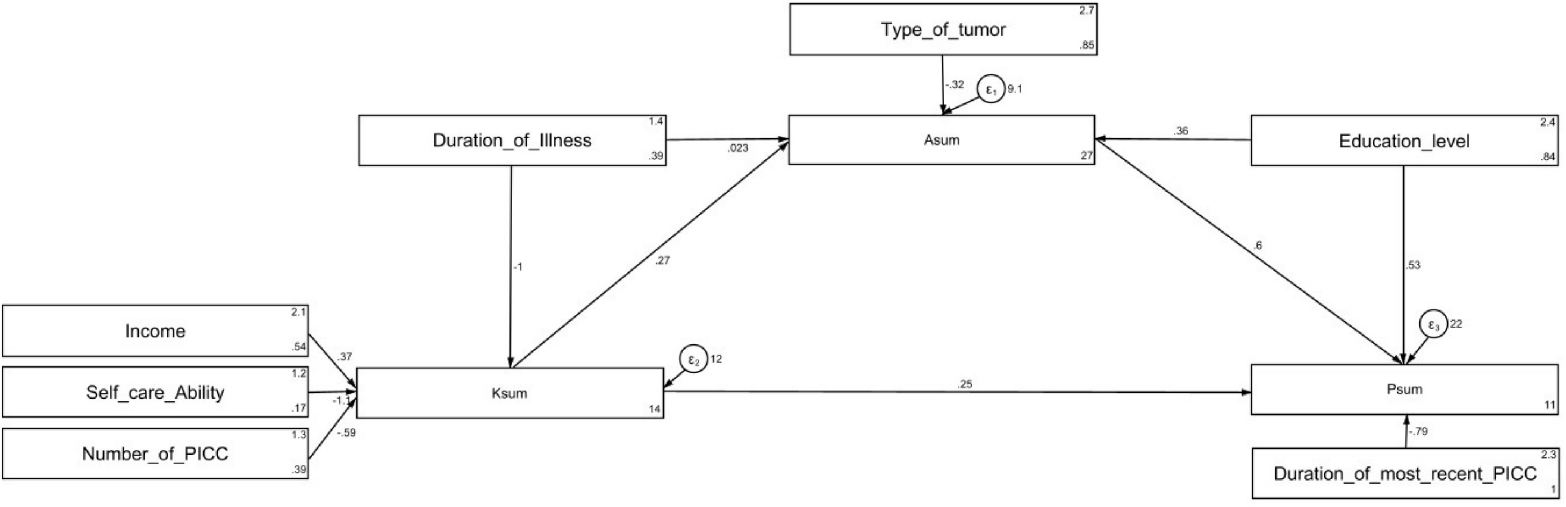
Path analysis.

Supplementary Table 2, Table 5, and Figure 1 present the path analysis. Path analysis demonstrated excellent model fit indices (RMSEA: 0.047, SRMR: 0.028, TLI: 0.891, CFI: 0.945), indicating a well-fitting model (Supplementary Table 2). Direct path analysis revealed that duration of illness (β = -1.04, *P* = 0.001) and self-care ability (β = -1.08, *P* = 0.01) directly influenced knowledge. Knowledge (β = 0.27, *P* < 0.001), education (β = 0.36, *P* = 0.021), and type of tumor (β = -0.31, *P* = 0.041) directly affected attitudes. Knowledge (β = 0.25, *P* < 0.001), attitudes (β = 0.59, *P* < 0.001), education (β = 0.52, *P* = 0.032), and the duration of the most recent PICC insertion (β = -0.79, *P* < 0.001) directly influenced practices. Additionally, duration of illness (β = -0.28, *P* = 0.002) and self-care ability (β = -0.29, *P* = 0.017) indirectly affected attitudes. Knowledge (β = 0.16, *P* < 0.001), education (β = 0.21, *P* = 0.026), type of tumor (β = -0.19, *P* = 0.047), duration of illness (β = -0.42, *P* = 0.032), and self-care ability (β = -0.45, *P* = 0.017) indirectly influenced practices (Table 5 and Figure 1).

**TABLE 5:** Direct and indirect effect of path analysis.

Model paths		Total effects		Direct effect		Indirect effect	
		β (95% CI)	*P*	β (95% CI)	*P*	β (95% CI)	*P*
**Asum**							
	Ksum	0.27 (0.19,0.34)	** < 0.001**	0.27 (0.19, 0.34)	** < 0.001**	–	–
	Education	0.36 (0.05, 0.67)	**0.021**	0.36 (0.05, 0.67)	**0.021**	–	–
	Monthly household income	0.09 (-0.02, 0.22)	0.128	–	–	0.09 (-0.02, 0.22)	0.128
	Type of tumor	-0.31 (-0.62, -0.01)	**0.041**	-0.31 (-0.62, -0.01)	**0.041**	–	–
	Duration of illness	-0.26 (-0.74, 0.217)	0.285	0.02 (-0.43, 0.48)	0.924	-0.28 (-0.46, -0.10)	**0.002**
	Level of self-care ability	-0.29 (-0.53, -0.05)	**0.017**	–	–	-0.29 (-0.53, -0.05)	**0.017**
	Number of PICC insertions you underwent	-0.15 (-0.33, 0.011)	0.068	–	–	-0.15 (-0.33, 0.011)	0.068
**Psum**							
	Asum	0.59 (0.45, 0.74)	** < 0.001**	0.59 (0.45, 0.74)	** < 0.001**	–	–
	Ksum	0.41 (0.28, 0.54)	** < 0.001**	0.25 (0.12, 0.37)	** < 0.001**	0.16 (0.10, 0.22)	** < 0.001**
	Education	0.74 (0.23, 1.25)	**0.004**	0.52 (0.04, 1.00)	**0.032**	0.21 (0.02, 0.40)	**0.026**
	Monthly household income	0.15 (-0.04, 0.34)	0.129	–	–	0.15 (-0.04, 0.34)	0.129
	Type of tumor	-0.19 (-0.38, -0.00)	**0.047**	–	–	-0.19 (-0.38, -0.00)	**0.047**
	Duration of illness	-0.42 (-0.80, -0.03)	**0.032**	–	–	-0.42 (-0.80, -0.03)	**0.032**
	Level of self-care ability	-0.45 (-0.82, -0.07)	**0.017**	–	–	-0.45 (-0.82, -0.07)	**0.017**
	Number of PICC insertions you underwent	-0.24 (-0.50, 0.019)	0.069	–	–	-0.24 (-0.50, 0.019)	0.069
	Duration of the most recent PICC placement	-0.79 (-1.22, -0.36)	** < 0.001**	-0.79 (-1.22, -0.36)	< 0.001	–	–
**Ksum**							
	Monthly household income	0.36 (-0.09, 0.82)	0.118	0.36 (-0.09, 0.82)	0.118	–	–
	Duration of illness	-1.04 (-1.64, -0.45)	**0.001**	-1.04 (-1.64, -0.45)	**0.001**	–	–
	Level of self-care ability	-1.08 (-1.91, -0.25)	**0.01**	-1.08 (-1.91, -0.25)	**0.01**	–	–
	Number of PICC insertions	-0.58 (-1.19, 0.020)	0.058	-0.58 (-1.19, 0.020)	0.058	–	–

Bold values represent statistically significant results (*P* < 0.05).

## 4. Discussion

Gastrointestinal cancer patients demonstrated sufficient knowledge, generally positive attitudes, and proactive practices regarding PICC management. Targeted educational interventions should be developed to further enhance patients’ knowledge and self-care abilities, particularly among those with longer illness durations and lower self-care capacities. These interventions should also aim to positively influence patients’ attitudes, which play a key mediating role between knowledge and practices, in order to optimize PICC care and reduce complications.

The findings of this study differ from previous research, such as a survey of critically ill cancer patients, which found that the KAP regarding PICC management after ICU discharge was unsatisfactory and in need of improvement (21). While our study showed relatively good KAP outcomes, there remain areas for improvement, particularly in knowledge and practices, which may explain the continued prevalence of PICC-related complications such as thrombosis. The adequate knowledge scores observed here may reflect patients’ understanding of PICC care. Indeed, at the study center, as per routine practice, the patients are taught PICC management based on the “*Expert Consensus on Venous Catheter Maintenance*” when they receive the PICC (17). However, certain gaps in practices remain, indicating the need for better patient education on managing PICCs independently (22). This suggests that deficiencies in nurse-led PICC care and education may contribute to the insufficient knowledge and practice scores of patients, as the support and guidance patients receive are critical to their ability to effectively manage PICC care. The generally favorable attitudes observed in this study indicate a willingness to engage in proper care practices, but the gaps in knowledge and practices highlight the need for targeted education to improve patient outcomes and compliance.

In terms of the relationships between KAP, correlation analyses, and path analysis results consistently demonstrated significant positive associations between knowledge, attitudes, and practices, indicating that higher levels of knowledge contribute to better attitudes and practices, which is supported by the literature (23, 24). For example, the correlation between knowledge and practices and the path coefficient of the path analysis both suggest that improving patients’ knowledge directly enhances their practical behaviors. Similarly, the significant influence of education on attitudes and practices highlights the role of education in fostering proactive care behaviors, a finding corroborated by other studies that emphasize the importance of patient education in healthcare settings (25). The path analysis also supports the direct impact of self-care ability and duration of illness on knowledge and practices, indicating that patients with longer illness durations and lower self-care capacities are at a higher risk of inadequate PICC management.

A particularly notable finding from our path analysis was the mediating role of attitudes in the relationship between knowledge and practices. The path analysis revealed that knowledge not only directly influenced practices but also indirectly affected practices through attitudes, with attitudes serving as a significant mediator. This finding aligns with the Health Belief Model, which suggests that health-related behaviors are influenced by individual beliefs and attitudes toward health conditions and their management (26). The mediating effect of attitudes suggests that simply increasing patients’ knowledge about PICC care may not be sufficient to improve their practices; rather, interventions should also focus on shaping favorable attitudes toward PICC management. This is consistent with previous research in chronic disease management, which has shown that patients’ attitudes and beliefs significantly mediate the relationship between knowledge and self-care behaviors (27). The strong mediating effect observed in our study (accounting for approximately 39% of the total effect of knowledge on practices) underscores the importance of addressing both cognitive and affective components in patient education programs.

When examining the distribution of KAP scores across variables such as education, duration of illness, and self-care ability, it is clear that patients with lower levels of education and self-care reported poorer outcomes. For example, patients with lower educational backgrounds were less likely to score highly in practice measures, a trend consistent with other studies suggesting that limited health literacy can negatively impact patient outcomes (28, 29). Similarly, those with longer illness durations exhibited poorer knowledge and practices, likely due to fatigue or diminished capacity for self-care. This is reflected in both the KAP scores and path analysis findings, where duration of illness had a significant negative effect on knowledge and an indirect negative effect on practices, further underscoring the importance of sustained educational interventions for chronic patients.

Given that the KAP scores were generally above 70%, the overall responses reflect a relatively positive outcome. However, targeted improvements are necessary in certain areas. Specific areas needing enhancement include patient understanding of thrombosis risks associated with hyperlipidemia, the causes of phlebitis, and proper PICC maintenance, such as catheter flushing and checking for damage. In the knowledge dimension, targeted interventions should focus on improving awareness about thrombosis and phlebitis, as these areas had the most frequent “unsure” responses in our study. Educational materials should emphasize the risks of thrombosis in patients with hyperlipidemia and explain how irritant medications can cause phlebitis. Additionally, hands-on workshops or multimedia resources can teach patients how to maintain their PICC by regularly flushing the catheter and checking for damage. These findings are consistent with similar studies that report knowledge gaps in PICC care (21). For the attitude dimension, concerns about proper PICC care and the perceived impact of PICC insertion on daily life were notable. Interventions should address patients’ anxieties regarding their ability to manage their catheter and the potential complications. Structured counseling or peer support programs could help build confidence in recognizing complications and managing PICC care independently. Hospitals should implement structured, ongoing educational programs that are tailored to patients’ educational backgrounds and self-care abilities (20, 30). In the practice dimension, issues such as avoiding activities like bathing or swimming, regularly checking for blood return, and inspecting the PICC for damage need attention. Educational initiatives should include clear, step-by-step guidance on safe PICC care practices, particularly regarding daily hygiene and catheter inspection. For instance, interactive workshops or mobile health platforms that provide regular reminders and information on clinical care have been shown to improve patient knowledge and adherence to care protocols (31–34). Healthcare providers should also implement individualized education plans, particularly for patients with longer illness durations or lower self-care abilities, to ensure that all patients receive the necessary support to manage their PICC effectively.

The present study aligns with and expands upon findings from the literature on KAP regarding PICC management in cancer patients. Multiple studies, including surveys of critically ill cancer patients post-ICU, revealed that while positive correlations exist among KAP domains, achievement of sufficient knowledge and satisfactory practice levels is inconsistent, often falling short and warranting improvement, especially in less-educated and long-term illness groups. Most studies emphasize that inadequate KAP is linked with higher rates of PICC-related complications and that individualized patient education is critical for effective catheter management (21, 35).

Compared to prior literature, which often described KAP levels as only moderate, the summary reflects relatively better KAP outcomes in gastrointestinal cancer patients, yet echoes common concerns about persistent gaps in knowledge and practice—particularly relating to thrombosis and phlebitis awareness. Both the summary and the literature highlight the important mediating role of attitude: educational interventions not only need to increase knowledge but also proactively shape attitudes, as these strongly mediate the translation of knowledge into proper practices. Path analysis findings in recent research, as described in the summary, are supported by other models like the Theory of Planned Behavior, which confirms that attitude and self-efficacy bridge the gap between what patients know and what they actually do regarding PICC care (36, 37).

A strong negative relationship between duration of illness and knowledge/practices among cancer patients with PICCs is counterintuitive, as longer illness duration might be expected to yield greater familiarity with catheter care through accumulated experience and education. However, research and clinical observations suggest several plausible interpretations for this finding. Indeed, long-term cancer patients often receive their initial education at the time of PICC insertion, but ongoing reinforcement of PICC care knowledge may be lacking, especially as clinical focus shifts to disease management and other priorities over time (38, 39). Many educational programs are front-loaded, and follow-up sessions tend to target newly diagnosed patients rather than those already enduring chronic illness. This can lead to gradual decline or stagnation in practical catheter care skills for long-term patients (40). Patients facing prolonged illness may experience care fatigue and psychological burnout, which can reduce motivation to maintain best practices and keep up-to-date with recommended PICC care protocols (38). Ongoing education and psychological support are essential for maintaining catheter care standards, but these components may be less emphasized for patients who are further along in their cancer journey (38). Health systems may have less structured protocols for periodic re-education about PICC maintenance, leading to informational gaps for long-term patients. There’s often a greater institutional focus on acute phases, new insertions, and complications, rather than routine reinforcement for established patients (21). Fragmented or poorly coordinated transitions between inpatient, outpatient, and home care can create discontinuities, making long-term patients more vulnerable to lapses in best practices (35). There is evidence that peer-led or digital education modules can effectively reinforce knowledge and practices among chronic patients, but their availability and uptake are uneven. Limited access can exacerbate disparities, particularly for those treated outside major metropolitan areas (38). Regular re-education and systematic reminders should be provided for all PICC patients, regardless of illness duration, ideally tailored to evolving needs and circumstances (38). Integrating psychological support and practical re-training in chronic phases may help counteract patient disengagement and knowledge attrition, improving long-term safety and outcomes (35, 38, 39). Therefore, the counterintuitive finding underscores the need for recurrent, adaptive education and support for long-term cancer patients with PICCs, and highlights potential risks when reinforcement is insufficient as the clinical course progresses. Nevertheless, patient training sessions could be included within events reserved for healthcare professionals. Likewise, healthcare professionals should also be trained to ensure patient empowerment. Some available studies demonstrate that this dual approach reduces complications, improves clinical outcomes, and increases both patient and staff confidence in vascular access care (41, 42).

Differences in treatment regimens and hospitalization duration among various tumor types can explain the variations in KAP scores observed in cancer patients with PICCs. Tumor type determines not only the intensity and duration of chemotherapy and related infusion therapies, but also the frequency of clinical contact, which shapes opportunities for education and reinforcement of best practices. Patients with gastrointestinal malignancies (e.g., colorectal, gastric) often undergo prolonged and intensive intravenous chemotherapy regimens, resulting in more frequent hospital visits and interactions with healthcare professionals, which enhances exposure to education and practical support for PICC care (43, 44). Other tumor types, such as those requiring shorter or less intensive regimens, may involve less time in clinical settings and fewer opportunities for reinforcement of proper PICC management, thereby impacting KAP scores (44). Tumor types that are associated with longer or repeated hospitalizations, often due to aggressive disease or therapy complications, allow patients greater exposure to structured education, monitoring, and hands-on assistance with PICC care (44, 45). Conversely, patients whose care is primarily outpatient or who experience shorter hospitalizations might receive less consistent reinforcement or education, which can negatively affect their KAP scores over time (44, 45). Therefore, tumor type can affect both KAP scores directly (through specific care requirements) and indirectly (through mediation by treatment regimen and hospitalization duration). These differences emphasize the need for regular, tailored patient education across tumor types and settings to ensure optimal knowledge and practice standards for PICC care (44, 45).

### 4.1 Strengths and limitations of the work

This study provides valuable insights into the relationship between KAP in PICC care among gastrointestinal cancer patients, identifying key areas for targeted educational interventions. By employing both correlation and path analysis, the study robustly examines how factors such as illness duration and self-care ability affect PICC management, offering guidance for personalized patient education. The finding that education exerts both direct and indirect effects on practice, mediated through knowledge and attitudes, represents a clear strength of the study and an actionable intervention point. Education programs for cancer patients with PICCs have robust evidence demonstrating improvements in self-management abilities, increased compliance, reduced complications, enhanced self-efficacy, and greater satisfaction with care (35, 38, 46).

However, several limitations should be noted. First, as the sample was from a single center, it is only partially representative of the broader population, with limitations in generalizability due to geographic, clinical stage, and care-setting constraints. This cohort provides useful insights into PICC use in gastrointestinal cancer patients from an urban-heavy, single-center population in China, but caution is required when extrapolating results to other regions with different socioeconomic and healthcare access profiles, other cancer types, and later clinical stages with longer disease duration, and settings with different care models, such as community oncology or home infusion services. For broader representativeness, a multicenter design encompassing various geographic regions, cancer types, and care settings would be needed to reduce selection bias and increase external validity. Second, self-administered questionnaires may have introduced response bias, as participants might have overestimated their knowledge and practices. Third, the cross-sectional design provides a snapshot of the study population at a precise moment in time, without the possibility of examining the impact of an intervention or evolution in time. It also prevents the establishment of causal relationships among variables and KAP dimensions. Lastly, as the study was conducted in a single cancer hospital, its findings may not fully generalize to other populations or healthcare settings.

### 4.2 Recommendations for further research

Future research should focus on conducting longitudinal studies to establish causal relationships between KAP regarding PICC management and patient outcomes. Expanding investigations to multiple healthcare settings and diverse patient populations will enhance the generalizability of findings, allowing for the adaptation of educational interventions to various contexts. Additionally, exploring the effectiveness of different educational formats, such as multimedia resources and peer support programs, could provide insights into optimal strategies for improving PICC care knowledge and practices.

### 4.3 Implications for Policy and Practice

The findings of this study highlight the need for structured, ongoing PICC education programs tailored to patients’ self-care abilities and illness durations, with particular attention to managing complications such as thrombosis and phlebitis. Healthcare policies should support patient-centered education initiatives in oncology, ensuring that all patients have access to comprehensive and easily understandable resources for managing their PICC care. Establishing standards for evaluating and enhancing KAP in PICC management will further contribute to improving the quality of care and patient outcomes across oncology settings.

## 5 Conclusion

Gastrointestinal cancer patients demonstrated adequate knowledge, positive attitudes, and proactive practices regarding PICC care. The observed correlations between knowledge, attitudes, and practices highlight the interconnected nature of these factors in patient care. These findings suggest that enhancing patient education, particularly targeting self-care ability and disease duration, could further improve PICC-related practices and overall patient outcomes.

## Data Availability

The original contributions presented in the study are included in the article/Supplementary material, further inquiries can be directed to the corresponding author.
