# Supplementary material for: Knowledge, attitudes, and proactive practices regarding PICC in gastrointestinal cancer: a mediation analysis of a patient empowerment attempt
Source: Front Med (Lausanne). 2026 Jan 5;12:1675258. doi: 10.3389/fmed.2025.1675258 (PMC12812651; doi:10.3389/fmed.2025.1675258)
Supplement: Supplementary file 1 [file Table_1.docx]

Questionnaire Number：

| Dear Participant：  We are researchers from ** Hospital and sincerely invite you to participate in our study. This research aims to understand patients' knowledge, attitudes, and practices regarding peripheral inserted central catheters (PICC) for gastrointestinal tumors, which will provide a basis for developing scientific intervention strategies. Your participation in this study is voluntary, and the study has been approved by the Ethics Review Committee. If you agree to participate, please review the following instructions:  1. Please complete the questionnaire. There are no right or wrong answers; just fill it out based on your actual situation. If you have any questions during the process, feel free to ask us, and submit the questionnaire promptly upon completion.  2. This study is a simple survey that will not harm your physical or mental health but may involve some personal privacy issues, such as your gender and age. We will strictly keep your information confidential and will not disclose it, so please fill it out with confidence.  3. As a participant, you can always inquire about information related to the study and its progress. If you decide to withdraw from the study, please inform us, and your data will not be included in the research results.  Thank you very much for taking the time to support our scientific research!  □I have been informed and agree to the use of the collected data for scientific research.  Informed Consent Signature：  Participation Date：      year      month      day | |
| --- | --- |
| **Part 1 basic information** | |
| 1. **Your age： years old** | |
| 1. **Your gender：** | a.male  b.female |
| 1. **Place of residence：** | a.urban  b.rural  c.suburban |
| 1. **Education level：** | a.primary school or below  b. middle school  c. high school/technical school  d. associated degree  e. bachelor’s degree  f. master’s degree or above |
| 1. **Your monthly household income：** | a.<2000  b.2000-5000  c.5000-10000  d.>10000 |
| 1. **Your type of tumor (select one)** | a. Esophageal cancer b. Gastric cancer c. Colorectal cancer d. Liver cancer e. Pancreatic cancer f. Bile duct cancer g. Other tumor |
| 1. **Duration of illness：** | a. <1 year b. 1-3 years c. 3-5 years d. >5 years |
| 1. **Your level of self-care ability:** | a. Fully self-care b. Partial self-care c. Unable to self-care |
| 1. **Number of PICC insertions you have had:** | a.1  b.2  c.≥3 |
| 1. **Duration of your most recent PICC placement:** | a. <1 month b. 1-3 months c. 3-6 months d. >6 months |

| **Part 2 Knowledge of PICC** | | | |
| --- | --- | --- | --- |
| 1. **PICC, also known as a Peripherally Inserted Central Catheter, is inserted through a peripheral vein into the central venous system.** | a.TRUE | b.FALSE | c.NOT SURE |
| 1. **PICC can be left in the blood vessel for a long time, facilitating continuous infusion and protecting the veins.** | a.TRUE | b.FALSE | c.NOT SURE |
| 1. **During bathing, protective film should be used to cover the PICC and it should be replaced promptly.** | a.TRUE | b.FALSE | c.NOT SURE |
| 1. **If redness, swelling, pain, and purulence occur at the puncture site after PICC placement, medical attention should be sought promptly.** | a.TRUE | b.FALSE | c.NOT SURE |
| 1. **Irritant medications may be one of the causes of phlebitis in PICC patients.** | a.TRUE | b.FALSE | c.NOT SURE |
| 1. **To prevent occlusion, PICC needs to be flushed with heparin weekly at the hospital.** | a.TRUE | b.FALSE | c.NOT SURE |
| 1. **If the catheter is obstructed, it should be removed directly.** | a.TRUE | b.FALSE | c.NOT SURE |
| 1. **To prevent catheter displacement, the arm with the PICC should avoid intense activities and external impact.** | a.TRUE | b.FALSE | c.NOT SURE |
| 1. **If the catheter becomes displaced, it can be adjusted by oneself.** | a.TRUE | b.FALSE | c.NOT SURE |
| 1. **When there is seepage or bleeding from the PICC site, no action is required.** | a.TRUE | b.FALSE | c.NOT SURE |
| 1. **PICC patients with hyperlipidemia are at risk of developing thrombosis.** | a.TRUE | b.FALSE | c.NOT SURE |
| 1. **To prevent thrombosis, gentle exercises such as fist clenching and stretching can be done with the arm that has the PICC.** | a.TRUE | b.FALSE | c.NOT SURE |
| 1. **Thrombosis can cause pain, swelling of the affected limb, and difficulty in movement.** | a.TRUE | b.FALSE | c.NOT SURE |
| 1. **The timing of PICC removal is determined by the doctor and should not be done by oneself.** | a.TRUE | b.FALSE | c.NOT SURE |
| 1. **If the catheter becomes displaced, it can be adjusted by oneself.** | a.FALSE | b.TRUE | c.NOT SURE |

| **Part 3 Attitudes Toward PICC** | | | | | |
| --- | --- | --- | --- | --- | --- |
| 1. **I believe that PICC can alleviate the pain of frequent injections associated with ordinary infusion.** | a.strongly agree | b.agree | c.neutral | d.disagree | e.strongly disagree |
| 1. **I believe that regular maintenance of the PICC is very important.** | a.strongly agree | b.agree | c.neutral | d.disagree | e.strongly disagree |
| 1. **I believe that maintaining personal hygiene is very important for PICC maintenance.** | a.strongly agree | b.agree | c.neutral | d.disagree | e.strongly disagree |
| 1. **I believe that avoiding intense activities helps prevent PICC displacement and displacement.** | a.strongly agree | b.agree | c.neutral | d.disagree | e.strongly disagree |
| 1. **I believe that PICC placement has affected my normal life.** | a.strongly agree | b.agree | c.neutral | d.disagree | e.strongly disagree |
| 1. **I am concerned that I might not be able to care for the PICC properly.** | a.strongly agree | b.agree | c.neutral | d.disagree | e.strongly disagree |
| 1. **I am confident in my ability to recognize PICC-related complications in a timely manner.** | a.strongly agree | b.agree | c.neutral | d.disagree | e.strongly disagree |
| 1. **I believe that the hospital should organize educational programs about PICC.** | a.strongly agree | b.agree | c.neutral | d.disagree | e.strongly disagree |

| **Part 4 Practice Toward PICC** | | | | | |
| --- | --- | --- | --- | --- | --- |
| 1. **I avoid taking baths or swimming while the PICC is in place.** | a.always | b.often | c.sometimes | d.occasionally | e.never |
| 1. **I regularly visit the hospital for PICC maintenance.** | a.always | b.often | c.sometimes | d.occasionally | e.never |
| 1. **I promptly replace the protective film if I notice it is curled, peeling, or loose.** | a.always | b.often | c.sometimes | d.occasionally | e.never |
| 1. **I avoid lifting heavy objects with the arm that has the PICC.** | a.always | b.often | c.sometimes | d.occasionally | e.never |
| 1. **I avoid compressing the catheter.** | a.always | b.often | c.sometimes | d.occasionally | e.never |
| 1. **I check the PICC daily for any damage.** | a.always | b.often | c.sometimes | d.occasionally | e.never |
| 1. **I check daily for blood return in the catheter.** | a.always | b.often | c.sometimes | d.occasionally | e.never |
| 1. **I actively learn about PICC catheterization knowledge.** | a.always | b.often | c.sometimes | d.occasionally | e.never |
