# Supplementary material for: Knowledge, attitudes, and proactive practices regarding PICC in gastrointestinal cancer: a mediation analysis of a patient empowerment attempt
Source: Front Med (Lausanne). 2026 Jan 5;12:1675258. doi: 10.3389/fmed.2025.1675258 (PMC12812651; doi:10.3389/fmed.2025.1675258)
Supplement: Supplementary file 2 [file Table_2.docx]

**Supplementary table 1. Correlation analysis**

|  | **Knowledge** | **Attitudes** | **Practices** |
| --- | --- | --- | --- |
| **Knowledge** | 1 |  |  |
| **Attitudes** | 0.2468 (P< 0.001) | 1 |  |
| **Practices** | 0.2432 (P< 0.001) | 0.4132 (P< 0.001) | 1 |

**Supplementary table 2. Path Analysis Fit**

| Indicators | Reference | Results |
| --- | --- | --- |
| RMSEA | <0.08 Good | 0.047 |
| SRMR | <0.08 Good | 0.028 |
| TLI | >0.8 Good | 0.891 |
| CFI | >0.8 Good | 0.945 |

Abbreviations :
‌RMSEA‌: Root Mean Square Error of Approximation

‌IFI‌: Incremental Fit Index‌

TLI‌: Tucker-Lewis Index

CFI‌: Comparative Fit Index
